# Genomic Evidence for the Recycling of Complex Organic Carbon by Novel *Thermoplasmatota* Clades in Deep-Sea Sediments

**DOI:** 10.1128/msystems.00077-22

**Published:** 2022-04-18

**Authors:** Peng-Fei Zheng, Zhanfei Wei, Yingli Zhou, Qingmei Li, Zhao Qi, Xiaoping Diao, Yong Wang

**Affiliations:** a Institute of Deep Sea Science and Engineering, Chinese Academy of Sciences, Sanya, Hainan, China; b State Key Laboratory of Marine Resources Utilization in the South China Sea, Hainan University, Haikou, China; c College of Life Science, Hainan Normal University, Haikou, China; d Institute for Ocean Engineering, Shenzhen International Graduate School, Tsinghua University, Shenzhen, China; Ocean University of China

**Keywords:** PAH, archaea, aromatic degradation, anaerobes, metagenome, dehalogenase, South China Sea

## Abstract

*Thermoplasmatota* have been widely reported in a variety of ecosystems, but their distribution and ecological role in marine sediments are still elusive. Here, we obtained four draft genomes affiliated with the former RBG-16-68-12 clade, which is now considered a new order, “*Candidatus* Yaplasmales,” of the *Thermoplasmatota* phylum in sediments from the South China Sea. The phylogenetic trees based on the 16S rRNA genes and draft genomes showed that **“***Ca.* Yaplasmales” archaea are composed of three clades: A, B, and C. Among them, clades A and B are abundantly distributed (up to 10.86%) in the marine anoxic sediment layers (>10-cm depth) of six of eight cores from 1,200- to 3,400-m depths. Metabolic pathway reconstructions indicated that all clades of “*Ca.* Yaplasmales” have the capacity for alkane degradation by predicted alkyl-succinate synthase. Clade A of “*Ca.* Yaplasmales” might be mixotrophic microorganisms for the identification of the complete Wood-Ljungdahl pathway and putative genes involved in the degradation of aromatic and halogenated organic compounds. Clades B and C were likely heterotrophic, especially with the potential capacity of the spermidine/putrescine and aromatic compound degradation, as suggested by a significant negative correlation between the concentrations of aromatic compounds and the relative abundances of clade B. The sulfide-quinone oxidoreductase and pyrophosphate-energized membrane proton pump were encoded by all genomes of “*Ca.* Yaplasmales,” serving as adaptive strategies for energy production. These findings suggest that “*Ca.* Yaplasmales” might synergistically transform benthic pollutant and detrital organic matter, possibly playing a vital role in the marine and terrestrial sedimentary carbon cycle.

**IMPORTANCE** Deep oceans receive large amounts of complex organic carbon and anthropogenic pollutants. The deep-sea sediments of the continental slopes serve as the biggest carbon sink on Earth. Particulate organic carbons and detrital proteins accumulate in the sediment. The microbially mediated recycling of complex organic carbon is still largely unknown, which is an important question for carbon budget in global oceans and maintenance of the deep-sea ecosystem. In this study, we report the prevalence (up to 10.86% of the microbial community) of archaea from a novel order of *Thermoplasmatota*, “*Ca.* Yaplasmales,” in six of eight cores from 1,200- to 3,400-m depths in the South China Sea. We provide genomic evidence of “*Ca.* Yaplasmales” in the anaerobic microbial degradation of alkanes, aliphatic and monoaromatic hydrocarbons, and halogenated organic compounds. Our study identifies the key archaeal players in anoxic marine sediments, which are probably critical in recycling the complex organic carbon in global oceans.

## INTRODUCTION

Recycling of organic matter in sediments is an important component of biogeochemical cycles because marine sediments are critical for long-term carbon storage (1, 2). Prokaryotes in deep oceanic subsurface account for more than 25% of global cell numbers (3), and it is still controversial whether *Archaea* or *Bacteria* are more abundant in this extensive ecosystem formed in marine subsurface sediments (4). Only few bacteria and archaea inhabiting marine sediment have cultivated representatives (5), which poses a major challenge when elucidating the metabolic mechanisms that sustain life, even abundant under the extreme energy-limiting conditions prevailing in the marine deep biosphere (6). Most groups of microorganisms inhabiting deep-sea subsurface sediment were proposed to be fueled by recalcitrant organic carbon (ROC) buried in the marine sediments (7). However, it remains unexplained how these subsurface microorganisms survive under the conditions where the available energy flux appears to be even 1,000-fold lower than the minimum value for maintenance energy estimated from laboratory cultures (6, 8). Considering the vast coverage of marine sediments on earth, our understanding of prokaryotic activities in subsurface marine sediment is still limited.

*Thermoplasmatota* is a globally distributed and ecologically important archaeal phylum that consists of several classes such as *Aciduliprofundales*, *Thermoplasmatales*, *Methanomassiliicoccales*, and “*Candidatus* Poseidoniales” (formerly called Marine Group II). These organisms were mostly found in surface seawater, while some clades are heterotrophic archaea in deep aphotic waters (9). “*Candidatus* Pontarchaea” (formerly called Marine Group III) and photic-zone MG-III metagenome-assembled genomes (MAGs) contained numerous photolyase and rhodopsin genes, as well as genes for peptide and lipid uptake and degradation, suggesting a photoheterotrophic lifestyle (10–12); *Thermoprofundales* (formerly called Marine Benthic Group D and DHVEG-1) was mixotrophic with detection of extracellular peptidase genes and Wood-Ljungdahl (WL) pathways (13). In a number of uncharacterized lineages, including UBA10834 (now referred to as “*Candidatus* Gimiplasmatales”), formaldehyde and acetate assimilation might proceed via the WL pathway, which indicates a mixotrophic lifestyle in sediment (14). As a novel clade in *Thermoplasmatota*, RBG-16-68-12 was first reported in terrestrial sediment at a 16-ft depth (15) and was also abundant in deep-sea sediment with water depth over 3,000 m (16). To date, there are few studies on RBG-16-68-12 archaea regarding their distribution depth and ecological function in nutrient-poor marine sediments. Compared to other archaeal phyla, studies on lineages of *Thermoplasmatota* are limited (12); a genome survey found that the *Thermoplasmatota* genomes contain the third highest number of unknown genes (17), suggesting that lineages of *Thermoplasmatota* should be further explored.

The particulate organic carbon (POC) export flux reached to 8.2 to 20 mmol of C m^−2^ day^−1^ in the South China Sea (SCS) shelf (18, 19). Recently studies showed that marginal seas, including the continental shelves of SCS, play a key role in the global carbon cycle by linking the terrestrial, oceanic, and atmospheric carbon reservoirs, which are effective in burying POC (20, 21). The POC provides abundant organic matter for microorganisms inhabiting the marine sediment. Among the abundant and prevalent organic matter in the environment, 16 polycyclic aromatic hydrocarbons (PAHs) as the major components of petroleum were declared as priority pollutants (22, 23). However, the roles of different microorganisms in organic matter processing in SCS continental slopes were not clear. In this study, we obtained MAGs of *Thermoplasmatota* dominated by RBG-16-68-12 from sediments of SCS. With analyses of 16S rRNA gene amplicons, we revealed prevalence of RBG-16-68-12 in >10 cm below seafloor (cmbsf) layers of six sediment cores. We could therefore confirm the independent phylogenetic position of RBG-16-68-12 consisting of three clades in *Thermoplasmatota*. Genomics data indicate metabolic potentials of aromatic and halogenated organic compound degradation, alkane utilization, and adaptive schemes to cope with energy limitation.

## RESULTS AND DISCUSSION

### Environmental parameters of the marine sediment samples.

**FIG 1:**
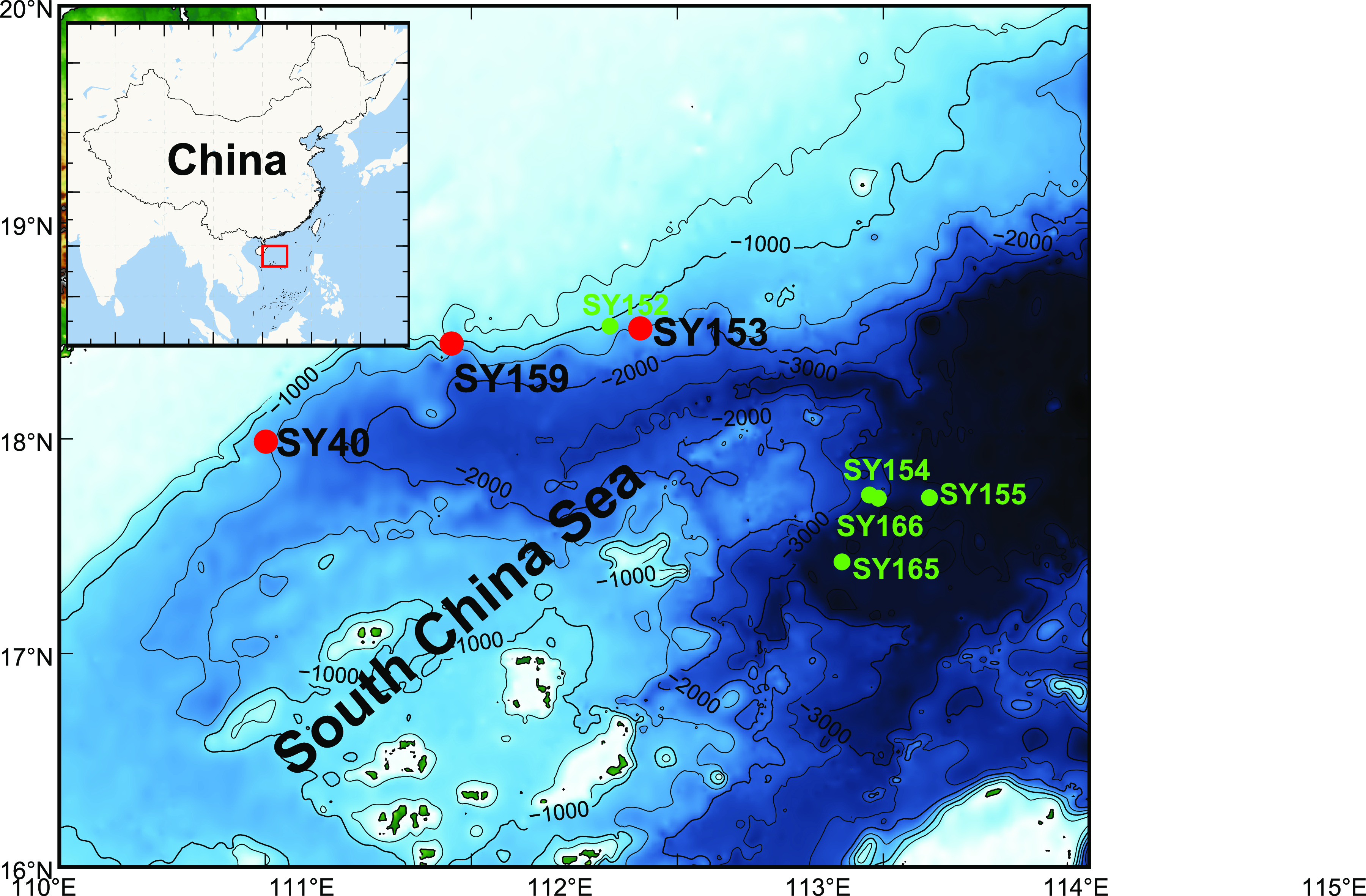
Sampling stations in the South China Sea. The sampling sites of sediment cores are indicated as red (for metagenomic study) and green (for 16S rRNA gene study) dots. All of the sediment cores were obtained by the *Deep-Sea Warrior* manned submersible with push core samplers. SY40, SY153, SY159, and SY152 were located on a marginal slope at the northwestern South China Sea at an ∼1,200-m depth (see Table S1 for more details).

We collected eight sediment cores (SY40, SY152, SY153, SY154, SY155, SY159, SY165, and SY166; Fig. 1) from the SCS continental shelf by the manned submersible *Deep-Sea Warrior*. Among the determined environmental parameters of the sediment cores, the concentrations of nitrate and nitrite in the surface (0 to 2 cm, nitrate: 2.55 to 16.45 μmol L^−1^; nitrite: 0.75 to 4.59 μmol L^−1^) were 2- to 3-fold higher than deeper sediment layers (8 to 24 cm, nitrate: 0.13 to 7.26 μmol L^−1^; nitrite: 0∼0.70 μmol L^−1^) (see Fig. S1 and Table S3 in the supplemental material). The ammonium concentrations in the different layers (0 to 18 cmbsf) were largely between 13.33 and 35 μmol L^−1^; the highest ammonium concentration of 81.48 μmol L^−1^ was measured in the deepest layer of SY159. Therefore, the deep sediment layers showed a higher level of ammonium compared to surface layers where nitrate concentration was highest. Similarly, silicate concentrations decreased with sediment depth, whereas phosphate concentrations did not vary with the depth (see Fig. S1). The phosphate concentrations in SY153 and SY159 were 5.51 to 6.77 μmol L^−1^, which were significantly higher than the 0.65 to 2.26 μmol L^−1^ in SY40 (*t* test; *P < *0.01). In contrast, the silicate concentrations in SY40 were significantly higher than those in SY153 and SY159 (Student *t* test; *P < *0.01) (see Table S3). We noticed that the TOC in our study ranged from 1.17 to 3.66%, which was consistent with previous reports (24, 25). The TOC/TN ratio as a frequently used index for organic matter origin was estimated to be 6.85 to 7.39 in SY153. Land plants and algae are diverse in their C/N ratios: marine algae, due to richness in protein and absence of cellulose, typically have C/N ratios between 4 and 10 (26, 27), and new lines of evidence suggested that cyanobacteria may produce more pentadecane (28). Therefore, the TOC/TN ratio of the sediments in this study indicates their origin from plants and algae.

10.1128/msystems.00077-22.1 FIG S1 Nutrients in sediment porewater in the SCS sediment samples. The sediment samples are listed in Table S3. Download FIG S1, TIF file, 0.6 MB. Copyright © 2022 Zheng et al. 2022 Zheng et al. https://creativecommons.org/licenses/by/4.0/ This content is distributed under the terms of the Creative Commons Attribution 4.0 International license.

10.1128/msystems.00077-22.7 TABLE S3 Environmental parameters of sediment samples in the SCS sampling sites. Download Table S3, DOCX file, 0.01 MB. Copyright © 2022 Zheng et al. 2022 Zheng et al. https://creativecommons.org/licenses/by/4.0/ This content is distributed under the terms of the Creative Commons Attribution 4.0 International license.

10.1128/msystems.00077-22.5 TABLE S1 Information of sampling sites. Download Table S1, DOCX file, 0.01 MB. Copyright © 2022 Zheng et al. 2022 Zheng et al. https://creativecommons.org/licenses/by/4.0/ This content is distributed under the terms of the Creative Commons Attribution 4.0 International license.

For the PAH measurements in our marine sediment of SY153, the anthracene was the most abundant PAH (460.63 ± 42.34 ng g^−1^, dry weight), followed by pyrene (436 ± 57.03 ng g^−1^, dry weight) and fluoranthene (239.89 ± 3.20 ng g^−1^, dry weight). The other PAHs were <100 ng g^−1^. The PAH measurement results in SY152 were similar to those in SY153, both much higher than the previous records for the total PAHs (170.7 to 498.2 ng g^−1^) (24). Among the different layers of SY152 and SY153, the most abundant PAHs were detected in surface layers (4 to 6 cmbsf and 8 to 10 cmbsf) and the deepest layer (24 to 26 cmbsf) (see Fig. S2). The ratio of phenanthrene and anthracene isomers in this study was 0.4 to 0.6, indicating a fossil fuel origin of the PAHs (29, 30).

10.1128/msystems.00077-22.2 FIG S2 Concentrations of 16 PAHs in sediment cores of SY152 and SY153. Download FIG S2, TIF file, 0.3 MB. Copyright © 2022 Zheng et al. 2022 Zheng et al. https://creativecommons.org/licenses/by/4.0/ This content is distributed under the terms of the Creative Commons Attribution 4.0 International license.

### Identification of MAGs for RBG-16-68-12.

We obtained 174 Gb clean metagenomic data for 12 sediment layers between 2 and 26 cmbsf of three push cores (SY40, SY153, and SY159) randomly selected from the eight cores (see Table S1). Metagenome assembly and genome binning resulted in a total of 201 bacterial and archaeal MAGs with a completeness of >50% and contamination at <10%. Five of the MAGs classified as *Thermoplasmatota* were obtained from the metagenomes for SY40 and SY153; among these, four MAGs were classified as RBG-16-68-12 by GTDB-tk software (31). As shown in Table 1, the genome size of the obtained *Thermoplasmatota* MAGs ranged from 1.37 to 2.03 Mb with a completeness between 61.84% and 98.93%.

**TABLE 1:** Genomic features of “*Ca*. Yaplasmales” and *Thermoprofundales*^*a*^

Category	Bin162	Bin292	Bin296	Bin295	Bin344
Classification	“*Ca.* Yaplasmales” clade A	“*Ca.* Yaplasmales” clade B	“*Ca.* Yaplasmales” clade B	“*Ca.* Yaplasmales” clade B	*Thermoprofundales*
No. of scaffolds	136	286	109	241	268
Genome size (Mb)	2.03	1.46	0.81	1.37	0.65
*N*_50_ value (kbp)	34.38	6.44	10.64	9.65	2.68
No. of protein coding genes	1,974	1,744	947	1,732	937
Completeness (%)	98.93	94.35	64.33	61.84	54.32
Contamination (%)	1.6	4.4	0	4.46	1.87
GC (%)	57.1	57.6	64.5	62.9	35.9
RED value	0.57	0.58	0.59	0.58	0.73
No. (%) of KEGG genes	1,049 (53.14)	1,030 (59.06)	593 (62.62)	792 (45.73)	523 (55.82)
No. (%) of COGs	1,395 (70.67)	1,267 (72.65)	723 (76.35)	1,051 (60.68)	638 (68.09)
Prevalence depth (cm)	25–26	16−18	16−18	16−18	22−24

a The five genomes were annotated in eggNOG database (v2020.08). Genome completeness and contamination were estimated using CheckM (v1.1.2). RED, relative evolutionary divergence.

**FIG 2:**
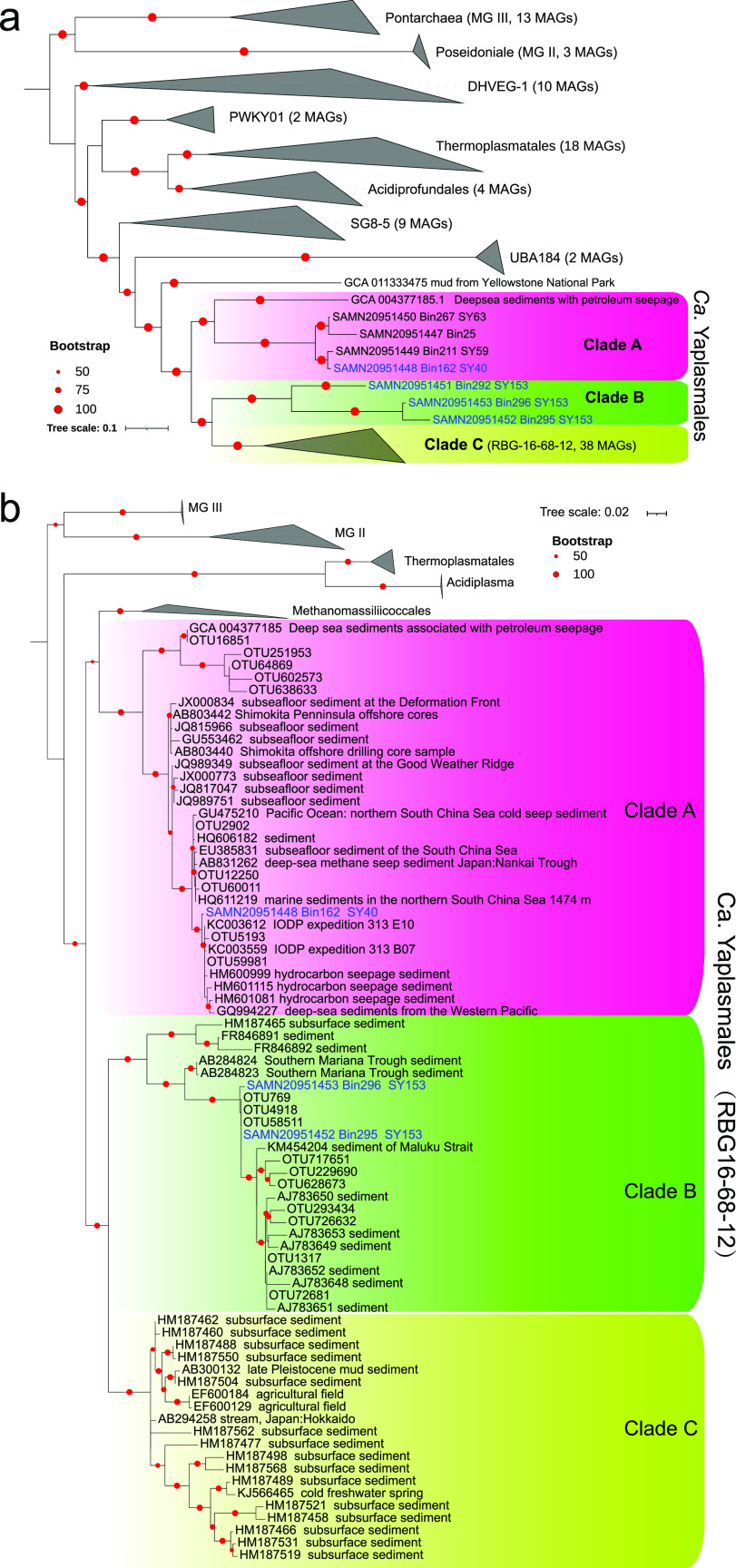
Phylogenetic trees of “*Ca.* Yaplasmales” (RBG-16-68-12). (a) An ML phylogenetic tree was constructed using alignment of concatenated 43 conserved proteins obtained from MAGs of *Thermoplasmatota*. The reference genomes of clades A and C are listed in Table S4. (b) A phylogenetic tree was constructed using 16S rRNA genes extracted from three “*Ca*. Yaplasmales” MAGs, representative sequences of OTUs from 16S rRNA amplicons, and the reference sequences within *Thermoplasmatota* from SILVA SSU database version 138 and NCBI. Bootstrap values were based on 1,000 replicates, and red solid nodes refer to the bootstrap values of >50%. The tree was drawn to scale, with branch lengths representing the number of substitutions per site.

Phylogenomic inference of the *Thermoplasmatota* genomes showed that the four MAGs of this study were grouped with members of RBG-16-68-12 (15, 32) adjacent to UBA10834 and SG8-5 (Fig. 2a). There are a total of 44 RBG-16-68-12 genomes in the NCBI database. In the tree, three distinguishable clades of RBG-16-68-12 were formed. The MAGs of the clades A and B were both obtained from deep-sea marine sediments with only one previously reported MAG. Therefore, we provide one new MAG in clade A and three new MAGs in clade B in this study. Four additional MAGs were obtained from SCS sediments by us for clade A and had been recently submitted to the NCBI. For the RBG-16-68-12 in NCBI database, 38 of 44 MAGs clustered into an independent clade C consisting of genomes largely from soil (*n* = 4) and temperate grassland microbiome (*n* = 31) (see Table S4). In addition, the average nucleotide identity (ANI) values among the RBG-16-68-12 MAGs obtained in this study were between 69 and 93% (see Table S5). The MAG Bin162 for clade A from SY40 was much different from those for clade B (Bin292, Bin295, and Bin296) in SY153 with ANI values of 69 to 70%, which is much lower than the values for a moderate affinity (ANI 80 to 90%) (33) and supports the divergency between the two marine clades. This result confirmed independence of RBG-16-68-12 clade as a novel lineage with more genomes, and we therefore propose “*Candidatus* Yaplasmales” as the new name of the lineage (34). The word (涯, “Ya”) in Chinese means limit, and the new clade name here indicates their survival in the deep-sea nutrient-limited sediments.

10.1128/msystems.00077-22.8 TABLE S4 Genomes of *Thermoplasmata* RBG-16-68-12 from GTDB. Download Table S4, DOCX file, 0.01 MB. Copyright © 2022 Zheng et al. 2022 Zheng et al. https://creativecommons.org/licenses/by/4.0/ This content is distributed under the terms of the Creative Commons Attribution 4.0 International license.

10.1128/msystems.00077-22.9 TABLE S5 ANI values for genomes of *Thermoplasmata* RBG-16-68-12 clades. Download Table S5, DOCX file, 0.01 MB. Copyright © 2022 Zheng et al. 2022 Zheng et al. https://creativecommons.org/licenses/by/4.0/ This content is distributed under the terms of the Creative Commons Attribution 4.0 International license.

The ANI values between *Methanomassiliicoccales* and “*Ca.* Yaplasmales” range from 67 to 70% (average, 69%), which suggests that they were close relatives. However, the analysis of 16S rRNA gene sequences showed that the similarity between *Methanomassiliicoccales* and clade A ranged from 87 to 89%, higher than the values between *Methanomassiliicoccales* and clade B (82 to 88%), indicating that the clades A and B are evidently different from *Methanomassiliicoccales*. Furthermore, the RED (the relative evolutionary divergence) value of the order *Methanomassiliicoccales* was 0.554, while the RED value of “*Ca.* Yaplasmales” lineage (RBG-16-68-12, GTDB-tk database, r202) was 0.528. This suggests that “*Ca.* Yaplasmales” was a new order of *Thermoplasmatota* (see Fig. S2).

### Prevalence of “*Ca.* Yaplasmales” in deep sediment layers.

The relative abundance of each “*Ca.* Yaplasmales” MAG in the three sediment cores was calculated as the percentage of the metagenomic reads that mapped to the MAGs. Our result showed that MAG Bin162 of clade A was only detected in 25 to 26 cmbsf of SY40 with 0.09% in relative abundance, and could not be found in all layers of the SY153 and SY159. The MAGs in clade B (Bin292, Bin296, and Bin 295) recruited 0.02 to 0.22% of metagenomic reads in 8 to 10 cmbsf and 16 to 18 cmbsf of SY153, respectively, which indicates a higher relative abundance of clade B in deep, possibly anoxic sediment layers (>10 cmbsf) compared to surface layers.

**FIG 3:**
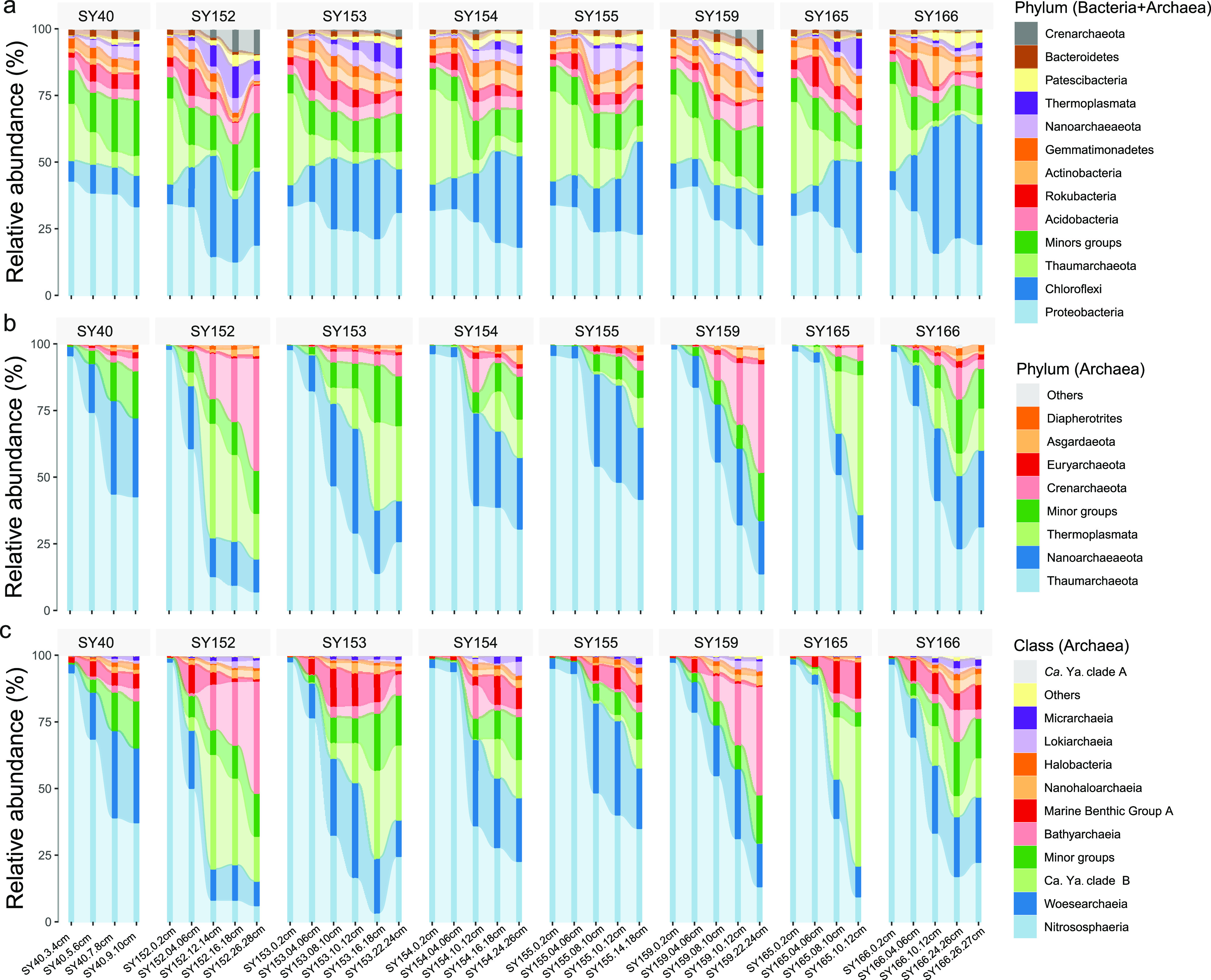
Distribution of “*Ca.* Yaplasmales” in eight sediment cores. (a) Prokaryotic community structures in eight sediment cores were revealed by sequencing and taxonomic sorting of 16S rRNA gene amplicons at the phylum level using SILVA 132 as a reference database. (b and c) Archaeal communities were separated to demonstrate their relative abundance at phylum (b) and class (c) levels. *Ca*. Ya. clade A, “*Ca*. Yaplasmales” clade A; *Ca*. Ya. clade B, “*Ca*. Yaplasmales” clade B.

To confirm the distribution of the “*Ca.* Yaplasmales” clades, we sequenced 16S rRNA gene amplicons in different layers of the eight sediment cores. Overall, 3,397,112 raw sequencing reads of 16S rRNA gene amplicons were obtained (see Table S2); of these, 2,914,782 qualified 16S rRNA gene sequences were used for operational taxonomic unit (OTU) sorting. The communities were dominated by *Proteobacteria*, *Chloroflexi*, and *Thaumarchaeota* (Fig. 3). Archaea accounted for 19.26% ± 5.96% of the whole microbial communities in the eight cores for this study (Fig. 2). For the archaeal community structure, *Thaumarchaeota* was the only dominant phylum in all sediment layers with an average relative abundance 56.27% of archaeal communities in 39 samples, and always occupied >90% of the archaeal communities in the top surface sediment (0 to 6 cmbsf) (Fig. 2). The relative abundance of *Thaumarchaeota* in all samples also decreased along with the sediment depth. Nitrososphaera, known as ammonia-oxidizing archaea (AOA), dominated the *Thaumarchaeota* in all the samples. *Nanoarchaeota*, *Thermoplasmatota*, and *Bathyarchaeota* were abundantly distributed in the deeper layers. The relative abundances of *Thermoplasmatota* were between 7.54% (SY159; 10 to 12 cmbsf) and 55.89% (SY165; 10 to 12 cmbsf) of the archaeal communities in the sediment samples over 10 cmbsf. In samples of SY152 and SY153, “*Ca.* Yaplasmales” clade B was much more abundant in >10 cmbsf than upper sample layers, since its relative abundance ranged from 15.29% (10 to 12 cmbsf of SY153) to 43.00% (12 to 14 cmbsf of SY152) of the archaeal communities (Fig. 2). Most (>50%) of *Thermoplasmatota* were assigned to “*Ca.* Yaplasmales,” and the rest were largely unclassified.

10.1128/msystems.00077-22.6 TABLE S2 Summary of metagenomic data and 16S rRNA gene amplicons. Download Table S2, DOCX file, 0.01 MB. Copyright © 2022 Zheng et al. 2022 Zheng et al. https://creativecommons.org/licenses/by/4.0/ This content is distributed under the terms of the Creative Commons Attribution 4.0 International license.

A phylogenetic tree was built with the 16S rRNA genes extracted from the MAGs, the 16S rRNA gene amplicons, and references from public databases. The phylogeny revealed more species in the “*Ca.* Yaplasmales” clades (Fig. 2b), some of which exhibit affinity to the sequences obtained from marine sediments and cold seeps located in SCS and the Gulf of Mexico. Clade C was exclusively composed of the sequences isolated from land soils.

The global distribution of “*Ca.* Yaplasmales” was examined by searching their 16S rRNA gene fragments in published data sets for a study of prokaryotic communities of 299 deep-sea sediment samples from 41 different sites (35). Within the published 16S rRNA amplicons, “*Ca.* Yaplasmales” were identified in 25 sediment samples from 14 sites with their relative abundance in prokaryotes almost all <0.1% (see Fig. S4). As expected, clade C was not detected in the marine sediments. Furthermore, “*Ca.* Yaplasmales” occupied 9.25% of the whole archaeal community (0.075% of the detected prokaryotes) in depth of 37.7m below seafloor of the Peru Basin (ODP leg 201, site 1231), which was the highest relative abundance among the 41 sites and was comparable to the highest abundance in this study. A sediment sample from the South Pacific Gyre (329U1367D1H-2) harbored >2% of the archaeal community. In brief, the distribution of “*Ca*. Yaplasmales” was wide in marine sediments, despite the low relative abundances. However, the usage of the different universal primers (U515F and U806R), compared to this study, in the published data sets might be causal to the low relative abundance.

10.1128/msystems.00077-22.4 FIG S4 Global distribution of “*Ca.* Yaplasmales” in 24 marine sediments libraries. “*Ca*. Yaplasmales” was identified in 16S rRNA amplicon-based archaeal communities in 24 marine sediment cores from global oceans (35). Their relative abundances are depicted as solid dots in different sizes and colors. Download FIG S4, TIF file, 0.5 MB. Copyright © 2022 Zheng et al. 2022 Zheng et al. https://creativecommons.org/licenses/by/4.0/ This content is distributed under the terms of the Creative Commons Attribution 4.0 International license.

### Metabolic potentials of “*Ca.* Yaplasmales.”

**FIG 4:**
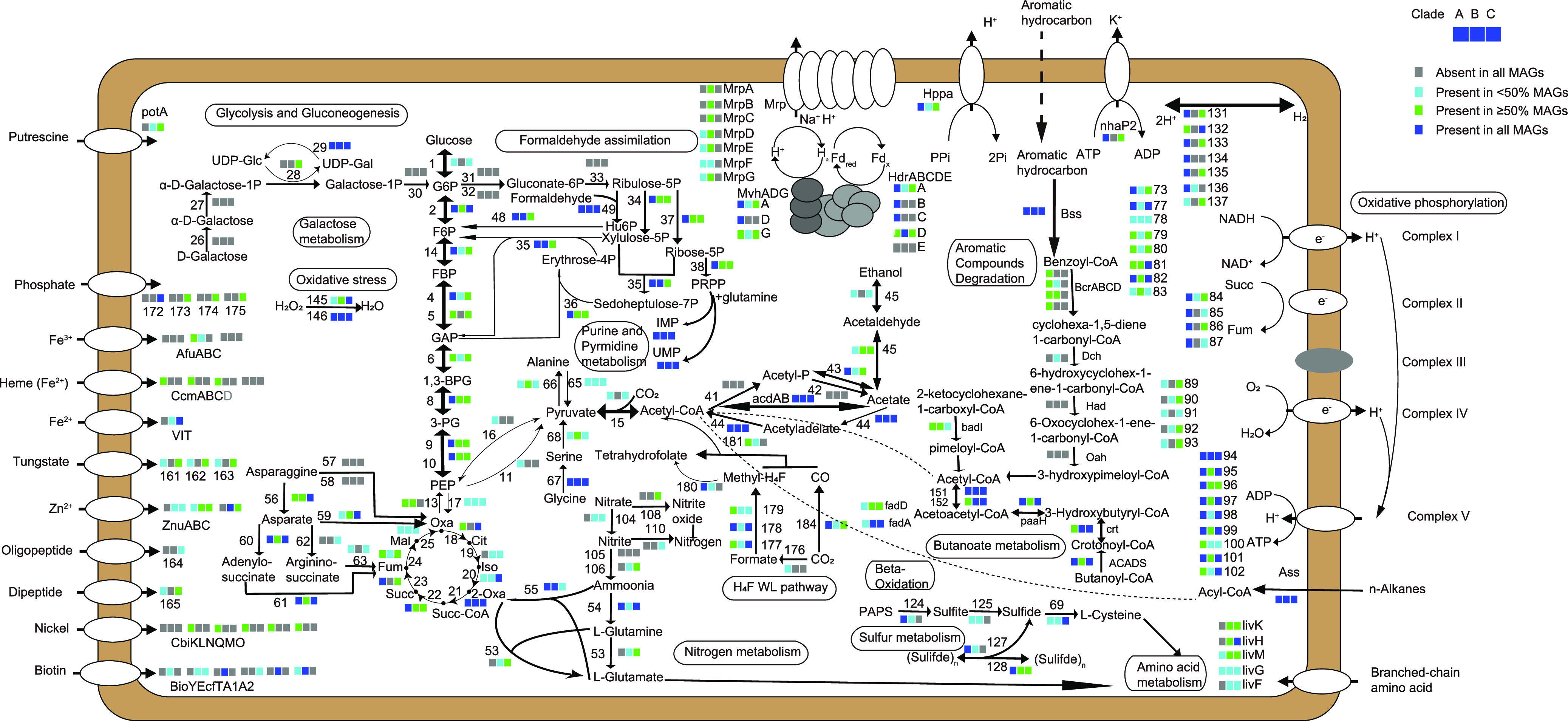
Predicted metabolic model of “*Ca.* Yaplasmales.” The three blocks next to the pathway steps and functional modules represent clades A, B, and C, and the different colors denote the percentages of MAGs that have the corresponding gene. The full names of genes with a number are presented in Table S6.

**FIG 5:**
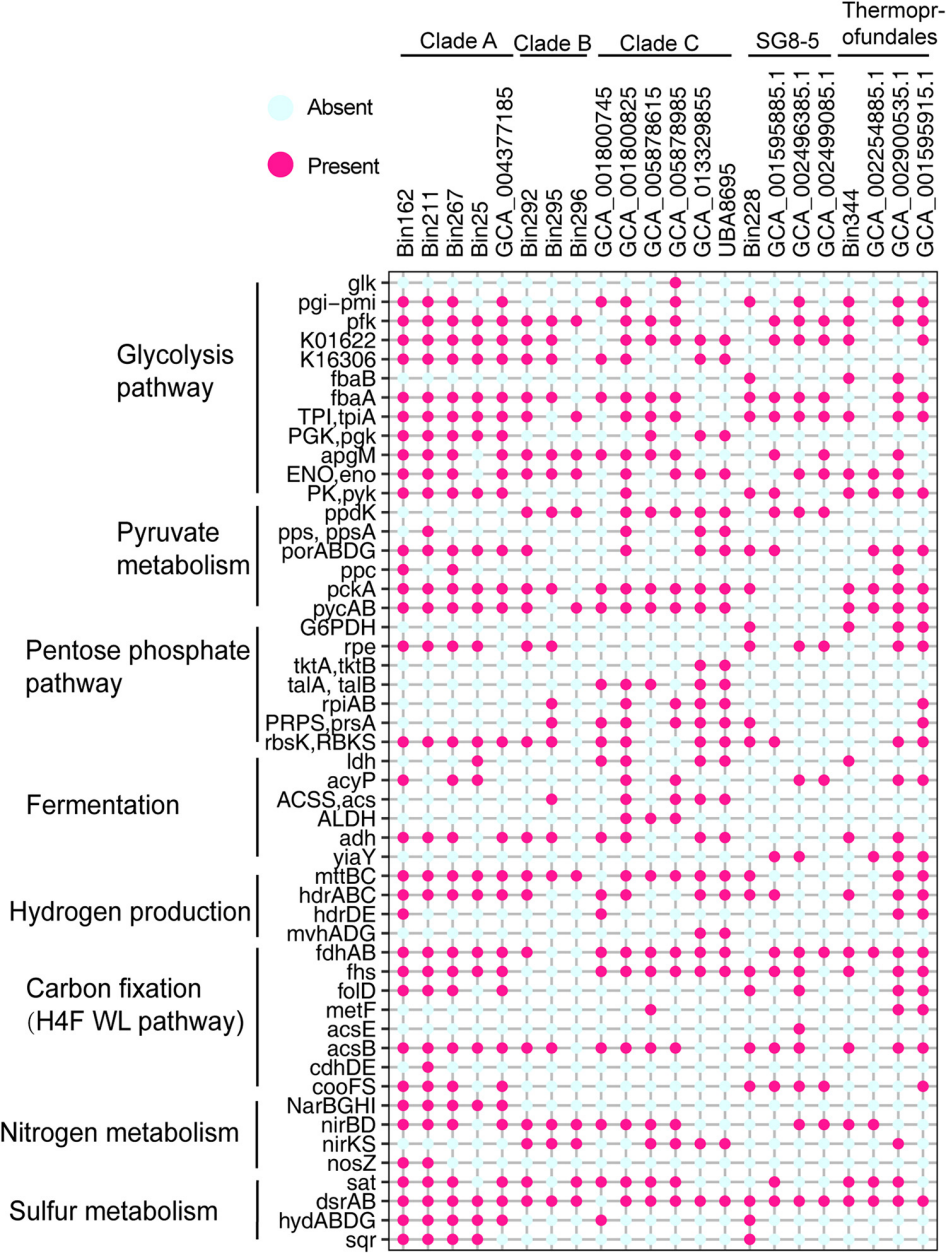
Comparison of functional genes in “*Ca.* Yaplasmales,” SG8-5, and *Thermoprofundales*. MAGs for “*Ca*. Yaplasmales” (clades A to C), SG8-5 clade, and *Thermoprofundales* were annotated against KEGG database. The copy number of functional genes are presented in Table S6.

The metabolic potentials of “*Ca.* Yaplasmales” clades were predicted based on annotation results of available MAGs affiliated with clades A and B, including nine reference MAGs from the NCBI and five from this study (Fig. 4). The six MAGs of clade C with a high completeness (2 MAGs > 97% and 4 MAGs >70%) were used for prediction of metabolic pathways. Based on the phylogenetic position of new class “*Ca.* Yaplasmales” (Fig. 3b), we compared their metabolic potentials to the closely related *Thermoplasmatota* in marine sediment inhabitants, such as *Methanomassiliicoccales*, SG8-5 and *Thermoprofundales* (Fig. 5). *Methanomassiliicoccales* was known for its methane metabolism (36), and *Thermoprofundales* was probably composed of mixotroph in mangrove and intertidal mudflat sediments (13). SG8-5 was inferred to have capability for extracellular protein degradation in sediment (37).

10.1128/msystems.00077-22.10 TABLE S6 List of genes and functions in Fig. 4 and 5. Download Table S6, XLSX file, 0.08 MB. Copyright © 2022 Zheng et al. 2022 Zheng et al. https://creativecommons.org/licenses/by/4.0/ This content is distributed under the terms of the Creative Commons Attribution 4.0 International license.

### (i) Carbon metabolism.

Alkane is ubiquitous carbon source for microbial inhabitants in marine sediment (38). 1-Methyl-alkyl-succinate synthase gene (*assA*) as a marker gene of anaerobic alkane degradation to acyl coenzyme A (acyl-CoA) (39, 40) was identified in all “*Ca.* Yaplasmales” MAGs, indicating their capacity of alkane utilization. The genes encoding acetyl-CoA acyltransferase (FadA) and long-chain acyl-CoA synthetase (ACSL and FadD) involved in further oxidation of acyl-CoA via the beta-oxidation were encoded in the MAGs. Therefore, alkanes are probably an important carbon source for all the clades of “*Ca.* Yaplasmales.” Genes encoding the bifunctional enzyme 3-hexulose-6-phosphate synthase/6-phospho-3-hexuloisomerase (HPS-PHI) were identified in all clades, suggesting the potential capacity in pentoses biosynthesis (41). The genes coding for acetate-CoA ligase (ADP-forming, AcdAB) and acetyl-CoA synthetase (Acs) were found in almost all “*Ca.* Yaplasmales” MAGs, which indicates that “*Ca.* Yaplasmales” might benefit from acetate assimilation or secretion. Acetate is an important energy and carbon source in marine sediment (42) and is also the central intermediate regulating community interactions and biogeochemical cycling in these deep-sea sediments (16). These inferences are in line with mounting evidence that acetogens are important community members in energy-limited seafloor ecosystems (42).

Regarding autotrophy, using a range of inorganic and organic substrates, the WL pathway produces acetate by acetogenic CO_2_ reduction (43) and was thought to be the largest carbon fixation pathway under anaerobic conditions (43, 44). Almost all of the MAGs from clade A harbor a complete set of WL genes, including the CO dehydrogenase (CooS and CooF) (45, 46) and acetyl-CoA synthase (CdhDE/AcsBE) genes (47) (Fig. 4; see also Table S6). The absence of WL pathway in Bin25 from clade A was likely due to its low genome completeness (60.67%), while all the MAGs from the clades B and C did not have a WL pathway (Fig. 4; see also Tables S4 and S8), as well as other known carbon fixation pathways. These results indicate that clade A has the genetic potential for inorganic carbon assimilation via the WL pathway for autotrophy, and clades B and C were likely heterotrophic archaea (Fig. 4; see also Table S6). The acetate metabolism of clade A might be associated with the WL pathway, which indicates the acetogenic capacity of this clade. Moreover, the WL pathway can probably be reversed and is associated with alkane degradation for CO_2_ production in marine sediment (48). Therefore, the WL pathway in this study is possibly a part of alkane degradation, rather than an autotrophic approach.

### (ii) Sulfur metabolism.

**FIG 6:**
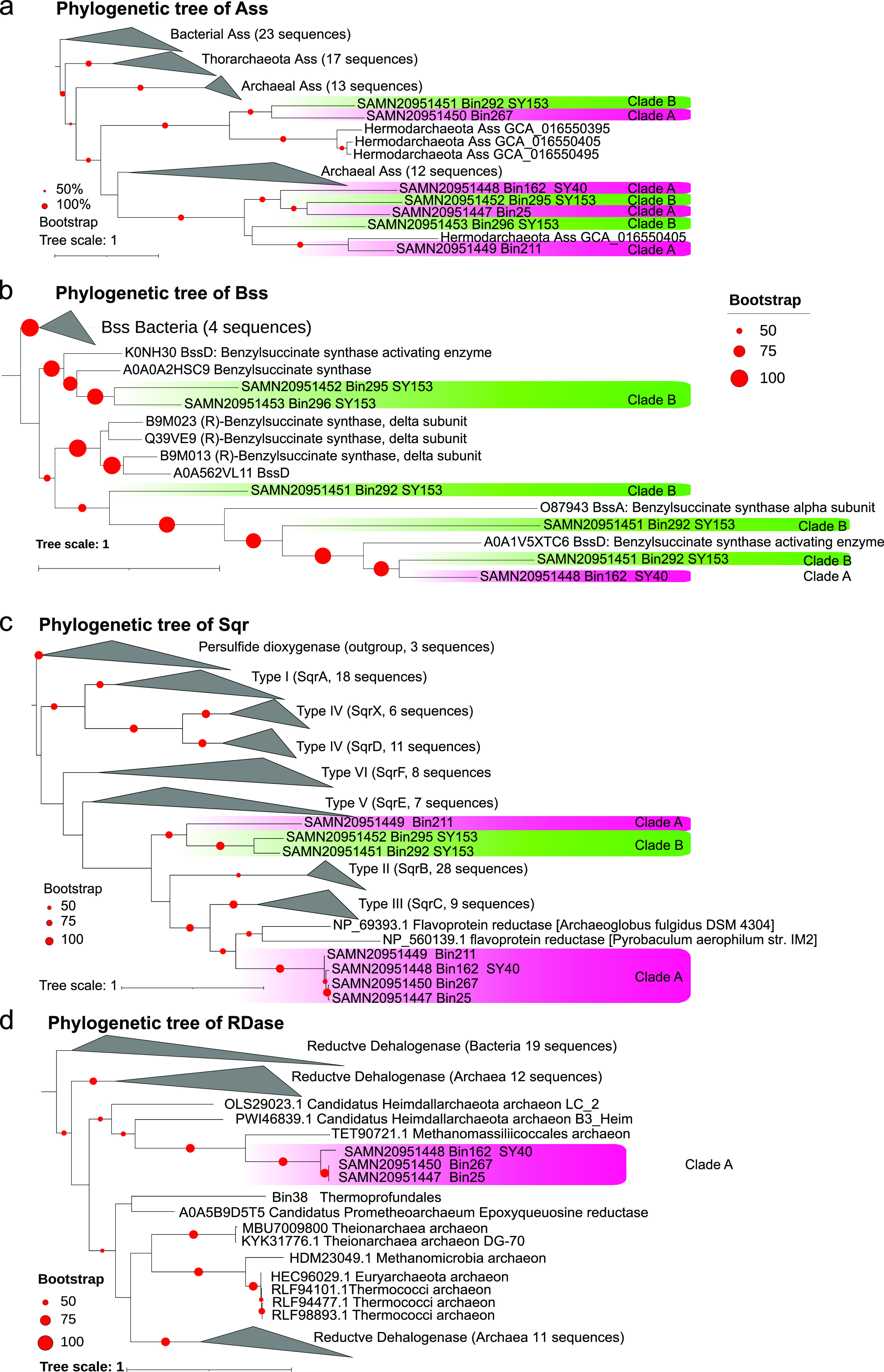
ML phylogenetic trees of the alkyl-succinate synthase (Ass), benzyl-succinate synthases (Bss), sulfide-quinone oxidoreductase (Sqr), and reductive dehalogenase (RDase) proteins in “*Ca*. Yaplasmales.” The phylogenetic trees of Ass (a), Bss (b), Sqr (c), and RDase (d) were inferred using MAFFT for alignment, and the ML tree was built using RAxML, with the number of bootstraps set to 1,000. Bootstrap values were denoted by dots on branches, and only those >50% were shown. The proteins from clades A and B are shaded with pink and green backgrounds, respectively.

The sulfide quinone oxidoreductase gene (*sqr*) responsible for sulfide oxidation was present in all the MAGs for clade A (Fig. 5 and 6c), and most of MAGs for clades B (2 of 3 MAGs) and C (4 of 6 MAGs) of “*Ca.* Yaplasmales,” which indicates that “*Ca.* Yaplasmales” might utilize sulfide at micro- to millimolar concentrations as an electron donor (49). Sqr is a membrane-associated protein that oxidizes sulfide to zero-valent sulfur and transfers electrons to flavin adenine dinucleotide (FAD). In particular, hydrogen sulfide biotransformation might also provide electrons, reduced FAD, and hydrogen to fix CO_2_ via WL pathway using H_4_-folate as C1 carriers for clade A. It is interesting that this enzyme in clade B is a potentially new type of Sqr with respect to the phylogenetics position (Fig. 6c) (50, 51).

### Degradation of halogenated organic compounds.

Halogenated compounds are widely reported in marine sediments as anthropogenic contaminants and natural products (52). From the Pfam annotation (53), we identified *rdh* gene encoding putative reductive dehalogenase (RDase) in clade A but not in clades B and C (Fig. 6d). The domain organizations of RDases are variable and fall into at least two categories: a C-terminal 4Fe-4S dicluster binding domain (Pfam13484) and a 4Fe-4S dicluster domain (Pfam12838) (54). In the present study, the RDases contain Pfam13484 or Pfam12838, which indicates that “*Ca.* Yaplasmales” clade A could metabolize halogenated organic compounds.

Halogenated organic compounds (organohalides) are a large class of natural and synthetic chemicals that contain one or more halogens (fluorine, chlorine, bromine, or iodine) combined with carbon and other elements, which are globally prevalent, recalcitrant toxic, and carcinogenic environmental pollutants (55). They are produced through abiotic and biotic processes in marine ecosystems and may serve as electron acceptors in deep-sea sediments (16). Reductive dehalogenation is a process that removes a halogen substituent from a molecule with concurrent reduction or adds two electrons to it, which is one of six enzyme-catalyzed dehalogenation reactions in microorganisms growing on halogenated organic compounds (56). It has been reported that some organisms can use halogenated substance as an energy source by transferring electrons to halogenated aromatic and aliphatic substances, a process termed organohalide respiration (57). The organohalide respiration pathway was one of the possible electron-acceptor systems in anoxic marine sediments (58, 59). Reductive dehalogenases are responsible for biological dehalogenation in organohalide respiring bacteria with a substrate source, not only from natural biological processes (59, 60) such as marine sponges and chlorinated biodegradation products of lignin but also some with an anthropogenic origin, including polychlorinated biphenyls or dioxins (60), and the results of this study indicate that “*Ca.* Yaplasmales” clade A plays an important role in recycling marine chlorinated pollutants (61).

The identification of alkyl-succinate synthase (Ass) and benzyl-succinate synthase (Bss; Fig. 6b) coding genes in clades A and B also suggests metabolic potentials of the degradation of alkanes and aromatic hydrocarbons. Bss catalyzes a highly unusual reaction: the addition of toluene to fumarate to form (*R*)-benzylsuccinic acid (62, 63), which drives anaerobic toluene degradation. Bss is now regarded as the prototype enzyme of a much larger family of fumarate-adding enzymes, which play important roles in the anaerobic metabolism of further aromatic and even aliphatic hydrocarbons (64). For the anaerobic oxidation of alkanes, an essential mechanism was also involved in the addition to fumarate, producing alkyl-substituted succinates (65), which was analogous to the anaerobic activation of aromatic hydrocarbons (66).

Furthermore, the *bcrABCD* genes in the class I ATP-dependent benzoyl-CoA reductase pathway were found in all clade A MAGs and in some MAGs in clades B (2/3 MAGs) and C (2/6 MAGs), which also indicates that “*Ca.* Yaplasmales” might utilize aromatic compounds (Fig. 4; see also Table S6) via a different approach. Benzoyl-CoA can be anaerobically degraded to yield a nonaromatic dienoyl-CoA intermediate by the *bcr* genes in anaerobes, including *Dehalococcoidia*, *Anaerolineae*, Deltaproteobacteria, *Bathyarchaeota*, and *Thermoplasmatota* (67).

The correlation analysis was performed between 16 PAH concentrations and “*Ca*. Yaplasmales” relative abundances (based on 16S rRNA gene amplicons), which showed a significantly negative correlation between clade B and anthracene (Pearson correlation; *R* = −0.69; *P < *0.05) and between clade B and phenanthrene (Pearson correlation; *R* = −0.78; *P < *0.01). This indicates that “*Ca*. Yaplasmales” clade B probably benefits from PAH degradation. The negative correlation between microorganism abundance and the phenanthrene concentration has been reported recently in cultivation (68). Hence, the distribution of “*Ca*. Yaplasmales” may be influenced by the anthracene and phenanthrene accumulation (see Fig. S3). The physical properties of PAHs, such as low aqueous solubility and high solid/water distribution ratios, affect their distribution in marine sediment, which may explain the low abundance of “*Ca.* Yaplasmales” in some of our cores (Fig. 3).

10.1128/msystems.00077-22.3 FIG S3 Taxonomic rank of “*Ca*. Yaplasmales” based on relative evolutionary divergence (RED). The RED values (*x* axes) are shown for all internal tree nodes with assigned taxonomic ranks (*y* axes). Rank assignments are based on the Genome Taxonomy Database (GTDB, r202). “*Ca*. Yaplasmales” clade A and B are indicated by red dashed lines. Download FIG S3, TIF file, 0.5 MB. Copyright © 2022 Zheng et al. 2022 Zheng et al. https://creativecommons.org/licenses/by/4.0/ This content is distributed under the terms of the Creative Commons Attribution 4.0 International license.

Archaea have been reported to mediate oxidation of methane and other short-chain alkanes in sediments (16, 69), whereas anaerobic degradation of larger and complex hydrocarbons (for example organohalides) is rarely reported. Recently, anaerobic alkane and aromatic compound degradation with alkyl-coenzyme A and benzoyl-coenzyme A oxidation were reported in a new archaeal phylum *Hermodarchaeota* (70). Similarly, the possible ability of “*Ca.* Yaplasmales” in degrading aromatic compounds in marine sediment may also benefit them with fitness. This is the first report of potential anaerobic aromatic degradation for *Thermoplasmatota*.

### Energy conservation mechanisms.

In the present study, hydrogenase genes were widely detected in “*Ca.* Yaplasmales” clades A and C (Fig. 5; see also Table S6). The encoded hydrogenases might supply intracellular reducing equivalents needed for various redox reactions and redox homeostasis (71). The MvhADG/HdrABC complex catalyzes an iron-sulfur cluster-assisted disulfide reduction reaction, which can be integrated into a flavin-based electron bifurcation (FBEB) process, a mode of energy coupling that optimizes the energy yield of the cell (72). The complex was frequently found in anaerobic bacteria and archaea (73), as well as in “*Ca.* Yaplasmales” MAGs (Fig. 4; see also Table S6), which indicated that the sediment layers dominated by “*Ca.* Yaplasmales” were probably oxygen limiting or anoxic. Here, the MAGs affiliated with clades A and C possessed the *hppA* gene that encodes K^+^-stimulated pyrophosphate-energized proton pump. HppA utilizes the energy of pyrophosphate hydrolysis as the driving force for proton movement across the membrane (74). The MAGs of “*Ca.* Yaplasmales” clade B harbor the *mnh* and *mrp* genes coding for multicomponent Na^+^:H^+^ antiporters as the proton or Na^+^ pumpers (Fig. 5), suggesting that clade B probably formed a functional complex involved in Na^+^ extrusion to maintain osmotic balance (75).

### Environmental adaptation of “*Ca.* Yaplasmales.”

As mentioned above, “*Ca.* Yaplasmales” archaea are probably anaerobes. The thioredoxin 1 (TrxA) that is able to protect anaerobes against oxygen (76, 77) was encoded by all “Ca. Yaplasmales” clades with up to five copies. TrxA plays a crucial role in maintaining the redox balance in various cells (78), with two thiol moieties that accept reducing elements from NADPH in reaction catalyzed by thioredoxin reductase (TR). The reduced TrxA can reduce disulfide bonds in proteins and helps them to maintain native structure or change conformation in response to various stimuli (79). The lack of the genes coding for a cytochrome *bc* complex and cytochrome *bd* terminal oxidase (Fig. 4; see also Tables S4 and S8) provides further evidence for anaerobic lifestyle of “*Ca.* Yaplasmales.” MAGs in clade A encode nitrate reductase/nitrite oxidoreductase complex (NarBGHI) and nitrite reductase (NirBD), which indicates capacity of nitrate utilization as electron acceptor for anaerobic respiration (Fig. 4; see also Table S6). Nitrite reductase coding genes *nirBD* and *nirKS* were also present in clades B and C MAGs, suggesting the two clades might reduce nitrite for respiration and detoxification.

Biotoxic arsenic oxides accumulate in marine sediment, which may impose environmental stress to microbial inhabitants (80). We found that almost all “*Ca*. Yaplasmales” MAGs (except Bin295 of clade B) harbored the arsenite methyltransferase gene (*arsM*) that is responsible to transformation of biotoxic arsenite to methylarsenite (81). The gene coding for arsenite transporter (ACR3) was also identified a few MAGs of different clades. Arsenate reductase (ArsC) was widely found in clade C (5/6 MAGs), but was rarely detected in clades A and B (Fig. 4; see also Table S6). This result indicates that “*Ca.* Yaplasmales” had the genetic potential to conduct biotransformation of arsenic oxides in the marine sedimentary environment (81).

### Conclusion.

In this study, we describe “*Ca.* Yaplasmales,” a novel order of *Thermoplasmatota* from deep-sea subsurface sediments collected from SCS. The metabolic potentials of all “*Ca.* Yaplasmales” clades indicated the utilization of sulfide oxidation in anoxic layers for energy and alkane and butanoate for a carbon source. The ecological importance of “*Ca.* Yaplasmales” is highlighted by its potential in the degradation of organohalides and aromatic compounds, which might significantly contribute to carbon and energy budgets in these deep-sea settings. Variations in the distribution of “*Ca.* Yaplasmales” in the deep-sea sites are probably accounted for by different carbon and sulfide sources in the sediments. Overall, this study expands our understanding of *Thermoplasmatota* by shedding light on its role in global element cycles and the biotransformation of marine pollutants.

## MATERIALS AND METHODS

### Sample collection.

Eight push cores with lengths of 24 to 26 cm were collected from eight different sites in the SCS (Fig. 1; see also Table S1) by the manned submersible *Deep-Sea Warrior* in dives 40, 152, 153, 154, 155, 159, 165, and 166 during R/V *Tansuoyihao* research cruises TS7 (March 2018) and TS12 (June and July 2019). Sampling sites were located in the Northwestern SCS slope at depths ranging from 1,200 to 3,400 m (Fig. 1; see also Table S1). Subsequently, each sediment core was segmented into sequential 2-cm layers in the shipboard lab at room temperature. The sediment subsamples obtained were immediately preserved in sterile tubes and frozen at −80°C for subsequent use.

### Environmental parameter and PAH measurements.

The sediment samples were centrifuged at 5,000 rpm for 10 min (Thermo Fisher, USA) to retrieve pore water (filtered through rinsed 0.45-μm-pore-size cellulose acetate membranes; Millipore, Germany) for the immediate analysis of environmental parameters. The concentrations of ammonium (NH_4_^+^), nitrate (NO_3_^−^), nitrite (NO_2_^−^), and soluble reactive phosphate (PO_4_^3−^) in the sediment pore waters were determined with a nutrient AutoAnalyzer (Seal, Norderstedt, Germany). The total organic carbon (TOC) and total nitrogen (TN) contents in the sediments were measured with a Vario Micro Cube elemental analyzer (Elementar, Langenselbold, Germany).

In order to measure the 16 priority PAHs in sediment, the wet sediment samples were processed by the freeze drying for 72 h to eliminate pore water, and then the grinded sediment was filtered with dichloromethane via a preconditioned solid-phase extraction C_18_ cartridge. After drying by nitrogen blowing, the resulting residue was redissolved with 1 mL of dichloromethane (82, 83). The analyses were performed on an Agilent 7890A GC (Agilent, USA) in the Analytical and Testing Center of Hainan University.

### DNA extraction and metagenome sequencing.

The total DNA of the sediment layer was extracted using a PowerSoil DNA isolation kit (MOBIO, Germany) according to the manufacturer’s instructions. The quality and quantity of genomic DNA and cDNA were checked by gel electrophoresis. DNA concentration was determined by using a Qubit dsDNA HS assay kit with a Qubit 2.0 fluorometer (Invitrogen, Carlsbad, CA). DNA samples with a low concentration of ≤2 ng/μL were concentrated using AMPure XP 546 beads (Beckman Coulter, CA) prior to library preparation. A total of 100 ng of DNA was randomly fragmented to ∼350 bp by Covaris M220 Focused ultrasonicator (Covaris, MA) using an Illumina TruSeq Nano DNA sample prep kit (Illumina, San Diego, CA). Libraries were sequenced on a Noveseq6000 platform to produce 2 × 150-bp paired-end reads (Illumina).

### Metagenomic assembly and genome binning.

Raw reads were trimmed to remove adapters and then filtered using fastp (version 0.20.0) (84) with parameters (-q 20 -u 40 -g -c -W 25 -3 -l 50). Low-quality reads (i.e., with a quality score of <20 for >40% of the length) and those shorter than 50 bp and unpaired were removed. The sequencing reads were then processed with fastuniq (v1.1) (85) to remove duplicated reads (see Table S2).

The qualified reads for each sediment layer were merged for assembly using Megahit (v1.2.8) (86) with a kmer range of 21 to 141 and k-step of 10 (–min-contig-len 300 -m 0.9 –k-min 21 –k-max 141 –k-step 10) to achieve the best assembly result. MetaWRAP v1.2.1 (87) was used for genome binning and subsequent refinement. The MAGs were checked by CheckM v1.0.11 (88) to filter those with low completeness (<50%) and high contamination (>10%). Taxonomic annotation of the MAGs was carried out using GTDB-tk software v0.2.2 (31). Then, the MAGs belonging to *Thermoplasmatota* archaea were retrieved for downstream analyses. The relative evolutionary divergence (RED) was calculated when a query genome could not be classified based on the ANI via GTDB-tk software.

### Genome annotation.

Reference *Thermoplasmatota* genomes were downloaded from the NCBI. Open reading frames of our MAGs and reference genomes were predicted by Prodigal (v 2.6.2) with option “-p meta” (89). The predicted genes were annotated by GhostKOALA online service (https://www.kegg.jp/ghostkoala, v2.2) and KofamScan (90) (version 1.1.0) against KEGG databases (91), by HMM search against protein families downloaded from Pfam v.34.0 (53), and by eggNOG-mapper v1.0.3 (92).

### Revealing microbial communities using 16S rRNA gene amplicons.

The V3–V4 region of 16S rRNA genes was amplified using a pair of universal primers: forward primer 341F (5′-CCTAYGGGRBGCASCAG-3′) and reverse primer 802R (5′-TACNVGGGTATCTAATCC-3′) (93, 94), with an 8-nucleotide barcode. The PCR system contained 1 ng of DNA as the template, 0.5 μL of each forward and reverse primer, respectively, 1 μL of HS DNA polymerase, 4 μL of dNTP mixture, and 10 μL of 5× PrimerSTAR buffer (TaKaRa, Dalian, China) in a 50-μL total reaction volume. The PCR products of partial 16S rRNA genes were purified using a Cycle-Pure kit (Omega, Norcross, GA) to degrade excess primers and nucleotides. An Illumina library for the equally pooled 16S rRNA gene amplicons was prepared by using a TruSeq Nano DNA LT kit (Illumina) and sequenced on an Illumina MiSeq platform.

The raw sequencing data of 16S rRNA gene amplicons were trimmed using an NGS QC Toolkit (v2.3.3) (95), and those with a low-quality score of <Q20 and shorter than 100 bp were discarded. The paired-end reads were assembled using multiple_join_paired_ends.py from QIIME (v1.9.1) with the default parameters. Afterward, the assembled reads were analyzed by using QIIME (v1.9.1) pipeline (96).

The 16S rRNA gene amplicons were clustered to the OTUs using UCLUST (v1.0.00) with a similarity threshold of 97% (97). The representative reads of the OTUs were used for further taxonomic classification with the SILVA 132 (98) database using UCLUST (97) at a similarity threshold of 97%. The OTUs assigned to mitochondria, chloroplasts, and eukaryotes were removed, and the singletons were also ignored. The RBG-16-68-12 OTUs were further confirmed by the phylogenetic relationships with the reference 16S rRNA genes and those extracted from the RBG-16-68-12 MAGs using a threshold similarity of 97% in BLASTN searching result.

### Phylogenetic analyses.

Phylogenetic analysis of 16S rRNA genes was performed with the sequences from the MAGs and NCBI-nt database, with 16S rRNA sequences of MG II and III groups from SILVA SSU database (version 132) serving as the outgroup. All 16S rRNA gene sequences were aligned with MAFFT v7.407 (99) with default setting, and sequences were trimmed via trimAl (v1.4) (100). Finally, a phylogenetic tree of 16S rRNA genes was constructed by the maximum-likelihood (ML) algorithm (GTRGAMMA model) using IQ-TREE 2 (-m MFP -B 1000 -alrt 1000 -T AUTO –bnni) (101).

Phylogenomic analysis of *Thermoplasmatota* genomes was conducted with 43 ribosomal proteins identified by CheckM program with default setting (88). Alignment of the obtained conserved protein sequences from the MAGs and reference sequences from NCBI SRA database was performed using MAFFT v7.407 (99) with the default settings, and poorly aligned regions were removed by trimAl (v1.4) (100). An ML phylogenomic tree was constructed using the concatenated aligned protein sequences with IQ-TREE tool (101) and the best-fit substitution model (LG+R10 model) for 1,000 replicates. In addition, all phylogenetic trees were visualized using the interactive Tree Of Life (iTOL) tool v6 (102).

### Genome abundance and prevalence.

The relative abundance of MAGs in the metagenomes was estimated by the recruitment rates of the qualified reads by BWA (103) and CoverM (with the settings -m relative_abundance -min-read-aligned-length 50 –min-read-percent-identity 0.99 –min-covered-fraction 0.1 –proper-pairs-only in genome mode) v0.6.1 (https://github.com/wwood/CoverM).

### Data availability.

The MAGs obtained from the marine sediment samples in this study have been submitted to the NCBI database under BioProject ID PRJNA757197.
