# Supplementary figures and images for: Genomic Evidence for the Recycling of Complex Organic Carbon by Novel Thermoplasmatota Clades in Deep-Sea Sediments
Source: mSystems. 2022 Apr 18;7(3):e00077-22. doi: 10.1128/msystems.00077-22 (PMC9239135; doi:10.1128/msystems.00077-22)

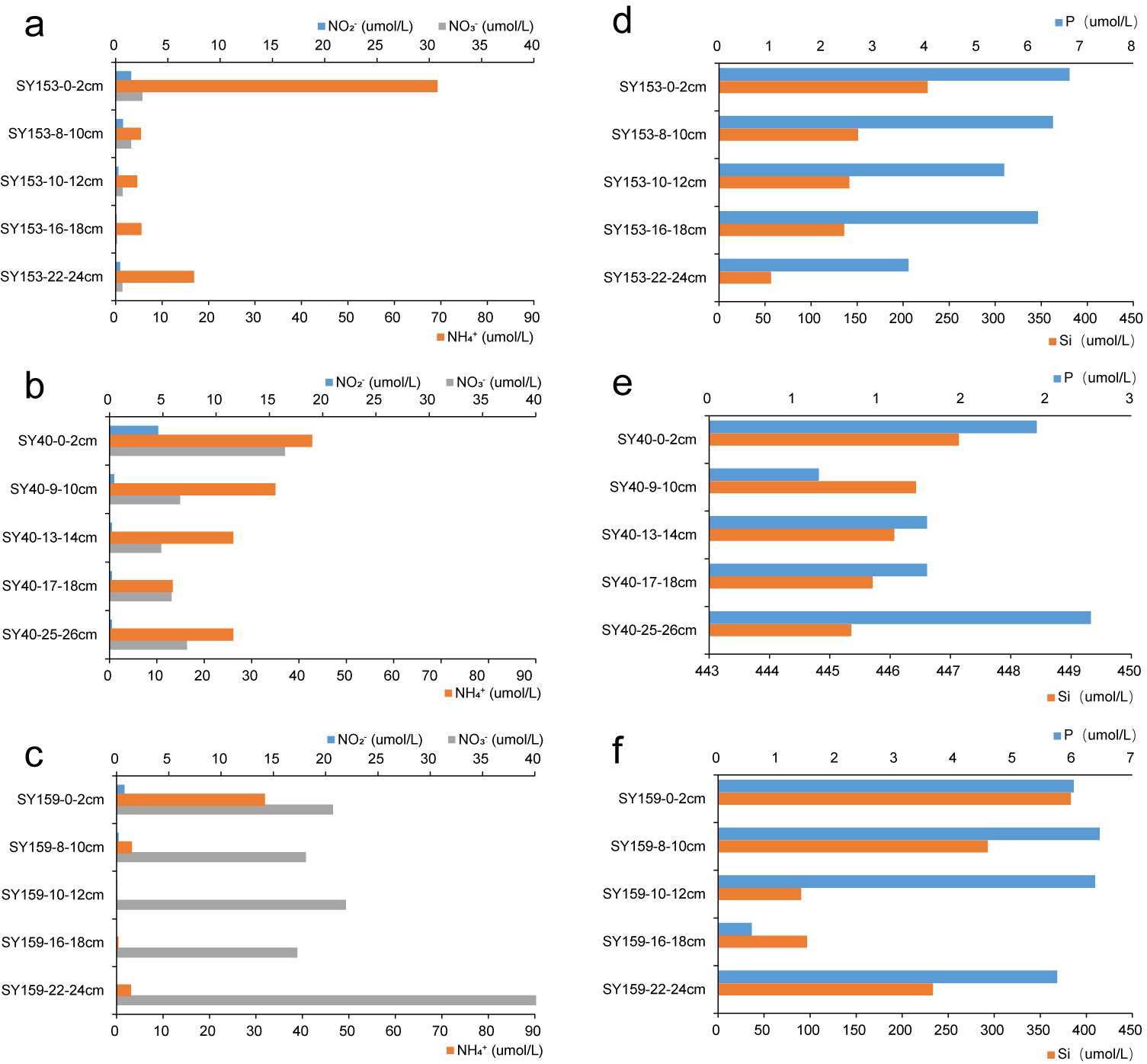

Supplement: FIG S1 [file msystems.00077-22-s0001.tif]

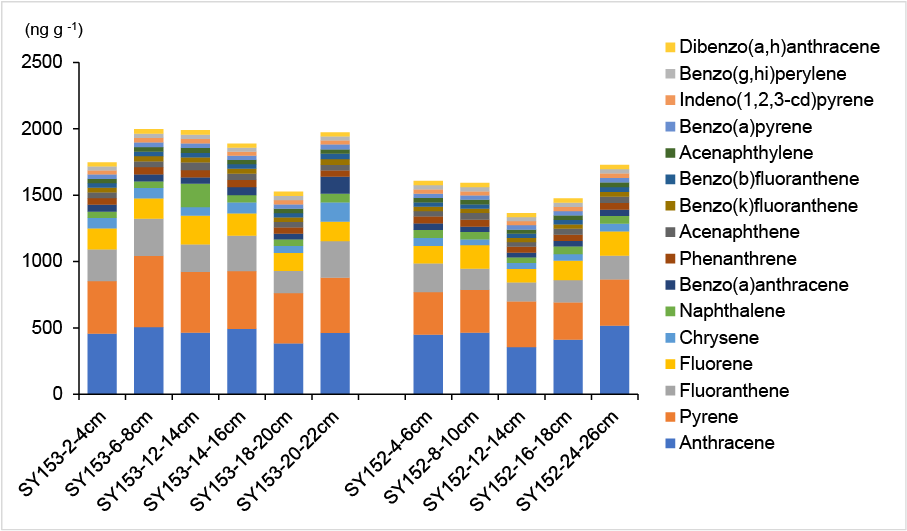

Supplement: FIG S2 [file msystems.00077-22-s0002.tif]

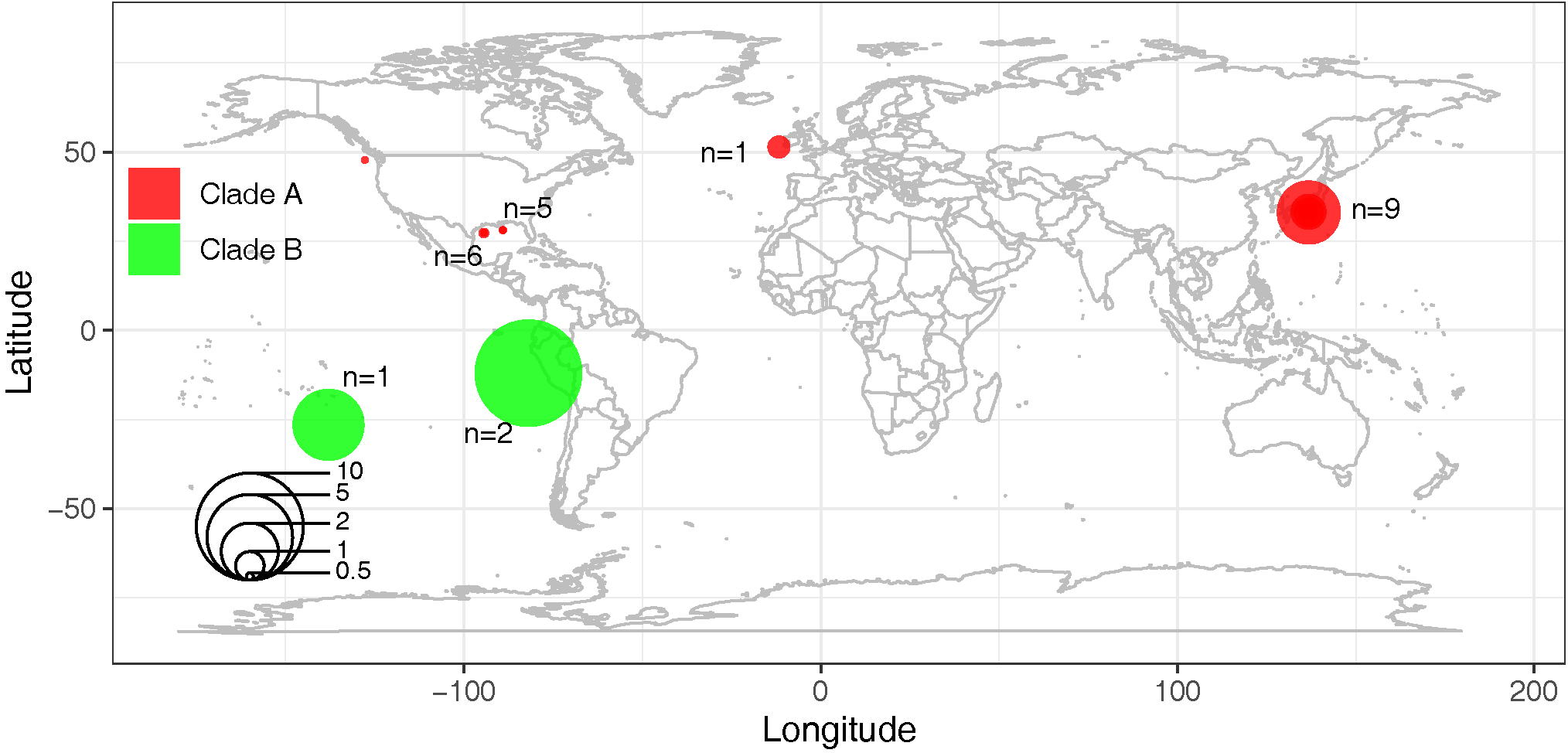

Supplement: FIG S4 [file msystems.00077-22-s0004.tif]

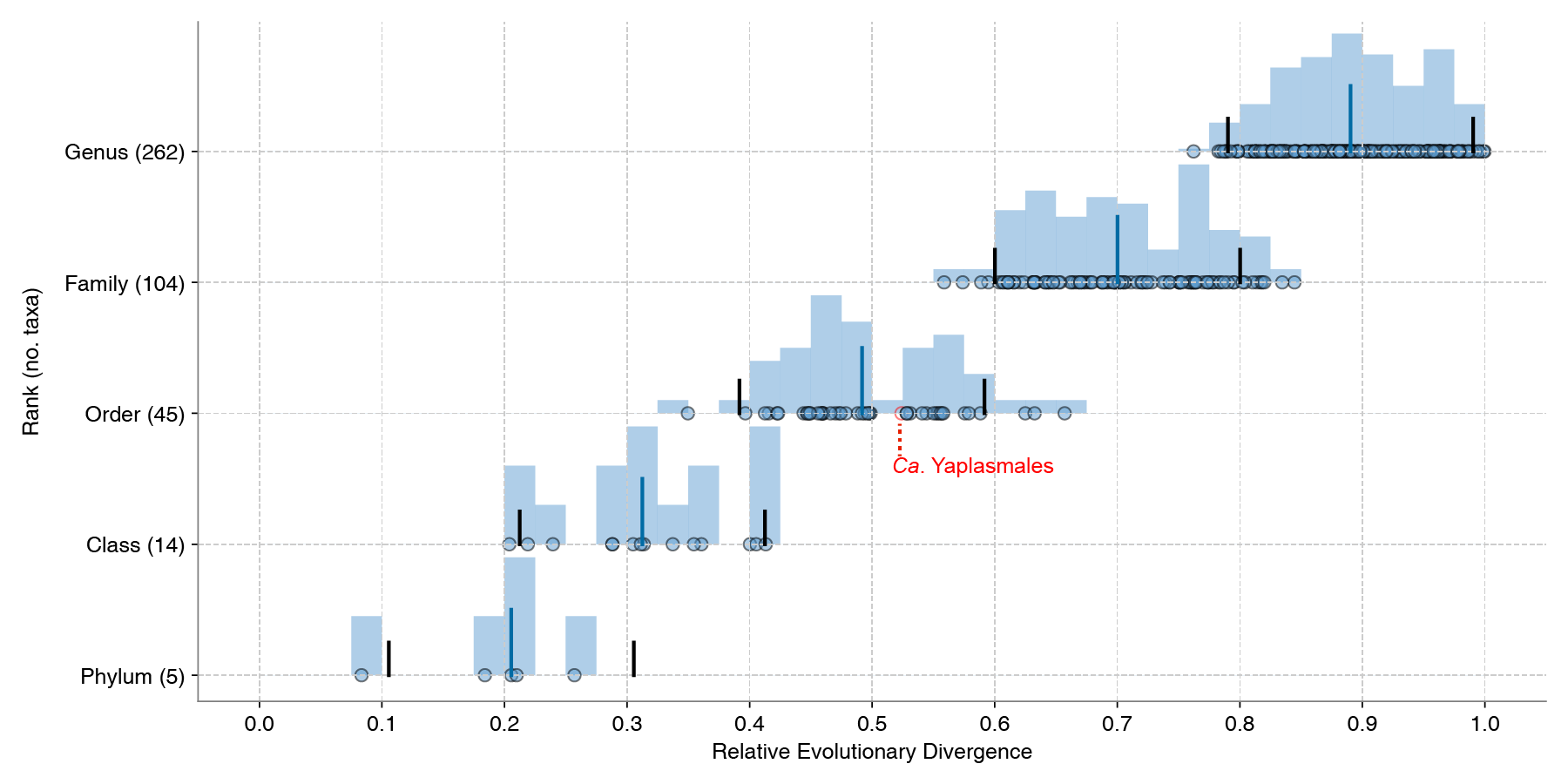

Supplement: FIG S3 [file msystems.00077-22-s0003.tif]
