# Supplementary material for: Genomic Evidence for the Recycling of Complex Organic Carbon by Novel Thermoplasmatota Clades in Deep-Sea Sediments
Source: mSystems. 2022 Apr 18;7(3):e00077-22. doi: 10.1128/msystems.00077-22 (PMC9239135; doi:10.1128/msystems.00077-22)
Supplement: TABLE S3 [file msystems.00077-22-s0007.docx]

Table S3 Environmental parameters of sediment samples in the SCS sampling sites.

|  | NO_2_^-^ (umol/L) | NH_4_^+^ (umol/L) | NO_3_^-^ (umol/L) | P（umol/L） | Si（umol/L） | TN (%) | TOC (%) | TOC:TN |
| --- | --- | --- | --- | --- | --- | --- | --- | --- |
| SY153-0-2cm | 1.49 | 16.85 | 2.55 | 6.77 | 56.67 | 0.23 | 1.60 | 6.85 |
| SY153-8-10cm | 0.70 | 5.56 | 1.48 | 6.45 | 136.00 | 0.15 | 3.66 | 24.63 |
| SY153-10-12cm | 0.27 | 4.63 | 0.67 | 5.51 | 141.67 | - | - | - |
| SY153-16-18cm | 0.10 | 5.43 | 0.14 | 6.16 | 151.11 | 0.14 | 1.27 | 9.23 |
| SY153-22-24cm | 0.43 | 69.26 | 0.65 | 3.66 | 226.67 | - | - | - |
| SY40-0-2cm | 4.57 | 26.11 | 16.45 | 1.94 | 445.36 | - | - | - |
| SY40-9-10cm | 0.43 | 13.33 | 6.61 | 0.65 | 445.71 | - | - | - |
| SY40-13-14cm | 0.22 | 26.11 | 4.84 | 1.29 | 446.07 | - | - | - |
| SY40-17-18cm | 0.22 | 35.00 | 5.81 | 1.29 | 446.43 | - | - | - |
| SY40-25-26cm | 0.22 | 42.78 | 7.26 | 2.26 | 447.14 | - | - | - |
| SY159-0-2cm | 0.75 | 20.69 | 3.11 | 6.01 | 233.50 | 0.20 | 1.43 | 7.29 |
| SY159-8-10cm | 0.19 | 18.10 | 0.37 | 6.45 | 96.73 | 0.16 | 1.17 | 7.18 |
| SY159-10-12cm | 0.06 | 21.93 | 0.13 | 6.37 | 90.29 | - | - | - |
| SY159-16-18cm | 0.00 | 17.28 | 3.30 | 0.57 | 293.02 | 0.16 | 1.26 | 7.68 |
| SY159-22-24cm | 0.00 | 81.48 | 31.90 | 5.73 | 383.33 | 0.16 | 1.19 | 7.39 |
