# Supplementary material for: Genomic Evidence for the Recycling of Complex Organic Carbon by Novel Thermoplasmatota Clades in Deep-Sea Sediments
Source: mSystems. 2022 Apr 18;7(3):e00077-22. doi: 10.1128/msystems.00077-22 (PMC9239135; doi:10.1128/msystems.00077-22)
Supplement: TABLE S1 [file msystems.00077-22-s0005.docx]

Table S1 Information of sampling sites

| **Sampling date** | **Cruise** | **Location** | **Latitude (N)** | **Longitude (E)** | **Depth (m)** | **MAG** | **Sampling date** | **Cruise** |
| --- | --- | --- | --- | --- | --- | --- | --- | --- |
| 2018.03 | TS07-1-Dive12 | SY40 | 17.017 | 111.000 | 1500 | Bin162 (clade a) | 2018.03 | TS07-1-Dive12 |
| 2019.06.28 | TS12-11 | SY153 | 18.009 | 112.014 | 1372 | Bin292 (clade b),Bin295 (clade b),Bin296 (clade b) | 2019.06.28 | TS12-11 |
| 2019.07.05 | TS12-11 | SY159 | 18.007 | 111.015 | 1526 | Bin344 | 2019.07.05 | TS12-11 |
| 2019.7.11 | TS12-11 | SY166 | 17.725 | 113.976 | 3316 | Thermoprofundales | 2019.7.11 | TS12-11 |
| 2019.7.10 | TS12-11 | SY165 | 17.428 | 113.799 | 1217 | Only 16S rRNA gene amplicons | 2019.7.10 | TS12-11 |
| 2019.6.30 | TS12-11 | SY155 | 17.727 | 114.224 | 3492 | Only 16S rRNA gene amplicons | 2019.6.30 | TS12-11 |
| 2019.6.29 | TS12-11 | SY154 | 17.739 | 113.933 | 3215 | Only 16S rRNA gene amplicons | 2019.6.29 | TS12-11 |
