# Supplementary material for: Genomic Evidence for the Recycling of Complex Organic Carbon by Novel Thermoplasmatota Clades in Deep-Sea Sediments
Source: mSystems. 2022 Apr 18;7(3):e00077-22. doi: 10.1128/msystems.00077-22 (PMC9239135; doi:10.1128/msystems.00077-22)
Supplement: TABLE S4 [file msystems.00077-22-s0008.docx]

Table S4 Genomes of Thermoplasmata RBG-16-68-12 from GTDB

| Clade | ID | %Completeness | %Contamination | Isolation source |
| --- | --- | --- | --- | --- |
| Clade A | GCA_004377185.1 | 97.6 | 1.6 | Deep sea sediments associated with petroleum seepage |
| Clade C | GCA_001800745.1 | 77.47 | 0.4 | Rifle background sediment; well D04 at 16ft depth |
|  | GCA_001800825.1 | 96.67 | 6.34 | Rifle background sediment; well D04 at 19ft depth |
|  | GCA_001800755.1 |  |  | Rifle background sediment; well D04 at 16ft depth |
|  | GCA_001800795.1 |  |  | Rifle background sediment; well D04 at 16ft depth |
|  | UBA8695 | 70.36 | 0 | Soil metagenome |
|  | GCA_013329855.1 | 70.36 | 0 | Soil metagenome |
|  | GCA_001919915.1 |  |  | Soil sample from Angelo meadow plot 1, 20cm depth |
|  | GCA_001920565.1 |  |  | Soil sample from Angelo meadow plot 1, 20cm depth |
|  | GCA_005878375.1 | 86.98 | 1.6 | Temperate grassland biome |
|  | GCA_005878915.1 | 89.16 | 4.4 | Temperate grassland biome |
|  | GCA_005878955.1 | 88.36 | 3.2 | Temperate grassland biome |
|  | GCA_005878415.1 | 77.74 | 3.93 | Temperate grassland biome |
|  | GCA_005878325.1 | 75.19 | 0 | Temperate grassland biome |
|  | GCA_005878635.1 | 81.55 | 2.4 | Temperate grassland biome |
|  | GCA_005878615.1 | 91.71 | 1.6 | Temperate grassland biome |
|  | GCA_005878385.1 | 84.98 | 0.56 | Temperate grassland biome |
|  | GCA_005879015.1 | 67.19 | 2 | Temperate grassland biome |
|  | GCA_005879065.1 | 87.6 | 0.8 | Temperate grassland biome |
|  | GCA_005878995.1 | 73.33 | 3.6 | Temperate grassland biome |
|  | GCA_005878485.1 | 90.45 | 1.6 | Temperate grassland biome |
|  | GCA_005878395.1 | 75.07 | 4.4 | Temperate grassland biome |
|  | GCA_005879045.1 | 80.46 | 3.2 | Temperate grassland biome |
|  | GCA_005878525.1 | 89.81 | 3.2 | Temperate grassland biome |
|  | GCA_005878665.1 | 79.6 | 2 | Temperate grassland biome |
|  | GCA_005878985.1 | 97.2 | 2 | Temperate grassland biome |
|  | GCA_005878515.1 | 82.13 | 3.42 | Temperate grassland biome |
|  | GCA_005878405.1 |  |  | temperate grassland biome |
|  | GCA_005878475.1 |  |  | temperate grassland biome |
|  | GCA_005878495.1 |  |  | temperate grassland biome |
|  | GCA_005878575.1 |  |  | temperate grassland biome |
|  | GCA_005878585.1 |  |  | temperate grassland biome |
|  | GCA_005878625.1 |  |  | temperate grassland biome |
|  | GCA_005878875.1 |  |  | temperate grassland biome |
|  | GCA_005878905.1 |  |  | temperate grassland biome |
|  | GCA_005878965.1 |  |  | temperate grassland biome |
|  | GCA_005879105.1 |  |  | temperate grassland biome |
|  | GCA_011331255.1 |  |  | temperate grassland biome |
|  | GCA_011362635.1 |  |  | temperate grassland biome |
