# Supplementary material for: Genomic Evidence for the Recycling of Complex Organic Carbon by Novel Thermoplasmatota Clades in Deep-Sea Sediments
Source: mSystems. 2022 Apr 18;7(3):e00077-22. doi: 10.1128/msystems.00077-22 (PMC9239135; doi:10.1128/msystems.00077-22)
Supplement: TABLE S5 [file msystems.00077-22-s0009.docx]

Table S5 ANI of genomes of Thermoplasmata RBG-16-68-12 clades

|  |  | Clade A | Clade B Clade B Clade B | | | Thermoprofundales |
| --- | --- | --- | --- | --- | --- | --- |
|  |  | Bin162 | Bin292 | Bin295 | Bin296 | Bin344 |
| Clade A | Bin162 | 1.00 | 0.69 | 0.69 | 0.69 | 0.77 |
| Clade B  Clade B  Clade B | Bin292 | 0.69 | 1.00 | 0.71 | 0.71 | 0.00 |
|  | Bin295 | 0.70 | 0.71 | 1.00 | 0.93 | 0.00 |
|  | Bin296 | 0.69 | 0.71 | 0.93 | 1.00 | 0.00 |
| Thermoprofundales | Bin344 | 0.73 | 0.70 | 0.00 | 0.00 | 1.00 |
