# Supplementary material for: Genomic Evidence for the Recycling of Complex Organic Carbon by Novel Thermoplasmatota Clades in Deep-Sea Sediments
Source: mSystems. 2022 Apr 18;7(3):e00077-22. doi: 10.1128/msystems.00077-22 (PMC9239135; doi:10.1128/msystems.00077-22)
Supplement: TABLE S2 [file msystems.00077-22-s0006.docx]

Table S2 Summary of metagenomic data and 16S rRNA gene amplicons.

| Samples | Layer (cmbsf) | Metagenomic sequencing data | | | | | 16S rRNA gene amplicons | |
| --- | --- | --- | --- | --- | --- | --- | --- | --- |
|  |  | Raw data | | Clean data | | MAGs number | Raw data no. reads | Clean data no. reads |
|  |  | total reads (M) | total bases (Gbp) | total reads (M) | total bases (Gbp) |  |  |  |
| SY40 | SY40_0-2 | 192.24 | 28.83 | 191.06 | 27.03 | 46 |  |  |
|  | SY40_3-4 |  |  |  |  |  | 52171 | 42713 |
|  | SY40_5-6 | 70.98 | 10.64 | 70.48 | 9.97 | 5 | 16625 | 13872 |
|  | SY40_7-8 | 19.74 | 2.94 | 19.74 | 2.78 | 1 | 40509 | 34959 |
|  | SY40_9-10 | 184.95 | 27.74 | 184.02 | 26.05 | 63 | 20130 | 17371 |
| SY153 | SY153_0-2 | 12.32 | 1.84 | 12.15 | 1.72 | 0 | 45787 | 39659 |
|  | SY153_4-6 | 119.94 | 17.99 | 119.14 | 16.88 | 11 | 48737 | 40153 |
|  | SY153_8-10 | 115.66 | 17.34 | 114.76 | 16.24 | 7 | 64885 | 54491 |
|  | SY153_10-12 |  |  |  |  |  | 56425 | 48740 |
|  | SY153_16-18 | 121.59 | 18.24 | 120.70 | 17.10 | 20 | 58652 | 50680 |
|  | SY153_22-24 |  |  |  |  |  | 64021 | 56701 |
| SY159 | SY159_0-2 | 119.01 | 17.85 | 118.39 | 16.77 | 8 | 28054 | 23891 |
|  | SY159_4-6 |  |  |  |  |  | 28781 | 24315 |
|  | SY159_8-10 | 93.26 | 13.98 | 92.82 | 13.14 | 12 | 29790 | 25396 |
|  | SY159_10-12 | 81.7 | 12.25 | 81.16 | 11.49 | 10 | 19120 | 16411 |
|  | SY159_22-24 | 105.09 | 15.76 | 104.57 | 14.83 | 18 | 29598 | 25897 |
